# A computationally efficient method for approximating reliabilities in large-scale single-step genomic prediction

**DOI:** 10.1186/s12711-022-00774-y

**Published:** 2023-01-05

**Authors:** Hongding Gao, Andrei A. Kudinov, Matti Taskinen, Timo J. Pitkänen, Martin H. Lidauer, Esa A. Mäntysaari, Ismo Strandén

**Affiliations:** Natural Resources Institute Finland (Luke), 31600 Jokioinen, Finland

## Abstract

**Background:**

In this study, computationally efficient methods to approximate the reliabilities of genomic estimated breeding values (GEBV) in a single-step genomic prediction model including a residual polygenic (RPG) effect are described. In order to calculate the reliabilities of the genotyped animals, a single nucleotide polymorphism best linear unbiased prediction (SNPBLUP) or a genomic BLUP (GBLUP), was used, where two alternatives to account for the RPG effect were tested. In the direct approach, the genomic model included the RPG effect, while in the blended method, it did not but an index was used to weight the genomic and pedigree-based BLUP (PBLUP) reliabilities. In order to calculate the single-step GBLUP reliabilities for the breeding values for the non-genotyped animals, a simplified weighted-PBLUP model that included a general mean and additive genetic effects with weights accounting for the non-genomic and genomic information was used. We compared five schemes for the weights. Two datasets, i.e., a small (Data 1) one and a large (Data 2) one were used.

**Results:**

For the genotyped animals in Data 1, correlations between approximate reliabilities using the blended method and exact reliabilities ranged from 0.993 to 0.996 across three lactations. The slopes observed by regressing the reliabilities of GEBV from the exact method on those from the blended method were 1.0 for all three lactations. For Data 2, the correlations and slopes ranged, respectively, from 0.980 to 0.986 and from 0.91 to 0.96, and for the non-genotyped animals in Data 1, they ranged, respectively, from 0.987 to 0.994 and from 0.987 to 1, which indicate that the approximations were in line with the exact results. The best approach achieved correlations of 0.992 to 0.994 across lactations.

**Conclusions:**

Our results demonstrate that the approximated reliabilities calculated using our proposed approach are in good agreement with the exact reliabilities. The blended method for the genotyped animals is computationally more feasible than the direct method when RPG effects are included, particularly for large-scale datasets. The approach can serve as an effective strategy to estimate the reliabilities of GEBV in large-scale single-step genomic predictions.

**Supplementary Information:**

The online version contains supplementary material available at 10.1186/s12711-022-00774-y.

## Background

Genomic selection can improve significantly the rate of genetic gain in animal breeding programs [1]. In particular, the use of single-step methods [2, 3] allows computing genomic estimated breeding values (GEBV) for both the genotyped and non-genotyped individuals simultaneously, even for large populations [4]. To assist selection decisions in practical breeding programs, the reliability of GEBV as a measure of the accuracy of the EBV can be provided along with the GEBV. In general, calculations of the reliability of GEBV use elements of the inverse left-hand side (LHS) matrix of the mixed model equations (MME) to obtain the prediction error variances (PEV). For large MME, inverting the LHS matrix is not computationally feasible, thus, approximated reliabilities are used.

Several methods to approximate the reliabilities of EBV from the pedigree-based best linear unbiased prediction (PBLUP) have been proposed and implemented [5–10]. In these methods, the first step is to account for only the most important non-genetic effects such as the contemporary group effects in the LHS matrix to approximate the effective record contributions (ERC) for the animals. In the second step, the ERC is combined with the pedigree-based relationship matrix information to calculate approximated reliabilities.

Misztal et al. [11] extended the PBLUP-based algorithm in [5] for the single-step method. They proposed two methods to approximate the reliabilities of GEBV from the single-step genomic BLUP (ssGBLUP). Their methods involved first approximating reliabilities by ignoring the genomic information, then converting those reliabilities into ERC for the genotyped animals, and subsequently obtaining the diagonal elements of the inverse of the LHS matrix of the MME in a genomic BLUP (GBLUP) model. Their methods are limited to the calculation of about 100,000 genotyped animals because the approach requires inverting the genomic and pedigree-based relationship matrices which are memory-intensive. The memory limitations can be overcome by using Markov chain Monte Carlo (MCMC) techniques and a single-step Bayesian regression model [12]. Gao et al. [13] demonstrated that exact PEV can be obtained by computing the posterior variance of GEBV samples, and then these PEV can be used to obtain the reliabilities of GEBV. However, MCMC is computationally demanding because it needs many iterations to obtain accurate PEV.

A multi-step procedure for approximating the reliabilities of GEBV was developed and introduced by Liu et al. [14]. Their method is based on a single nucleotide polymorphism BLUP (SNPBLUP) model with the polygenic effect accounted for in the calculation of the reliabilities of GEBV for the genotyped animals. The genomic information was propagated to the non-genotyped animals via ERC. Edel et al. [15] developed an approach using a single-step SNPBLUP model where genotypes for the non-genotyped animals are imputed from the observed genotypes with a reduced pedigree. The PEV matrix of the marker effects is based on all markers (observed and imputed) taking the imputation residuals into consideration. Recently, Bermann et al. [16] presented a method to approximate the reliabilities of GEBV using the Algorithm for Proven and Young (APY) [17] and showed that, with the reduced dimensionality, their approach can approximate reliabilities for a large population. Ben Zaabza et al. [18] used a similar approach as Liu et al. [14], in which a weighted GBLUP or SNPBLUP model with the inclusion of the extra information from the non-genotyped animals was used to compute the reliabilities of GEBV for the genotyped animals. For the non-genotyped animals, they used a procedure to integrate the genomic information gained from the genotyped animals in terms of the additional ERC into the conventional PBLUP model.

SNPBLUP and GBLUP are equivalent models of which either one can be used to compute the PEV needed for the reliabilities. The most significant advantage of the marker-based model such as SNPBLUP, is the constant dimension of the MME with respect to the number of genotyped animals because the size of the MME depends only on the number of markers. Therefore, with an increasing number of genotyped animals, the SNPBLUP model is preferred over the GBLUP-based model that uses the genomic relationship matrix. However, when the RPG effect is included in the SNPBLUP model, this advantage will no longer hold. The inversion of the LHS matrix of the MME, with more genotyped animals, becomes computationally demanding with both GBLUP and SNPBLUP. Attempts have been made [19, 20] to use Monte Carlo (MC) sampling to calculate the reliabilities of GEBV using SNPBLUP with RPG, but the MC methods can be too demanding computationally as many MC samples for traits having a high proportion of RPG effects are required. Hence, methods that are computationally efficient for the calculation of reliabilities when using a SNPBLUP or a GBLUP model with the RPG effects are needed.

A document distributed by Interbull in 2018 on the calculation of effective daughter contributions (EDC) of bulls and ERC for cows (https://interbull.org/static/web/A_supplementary_document_to_the_Interbull_genomic_reliability_method-1.pdf), can be used to compute the weights for animals compared to deregressing the reliability values. This method has been implemented by all countries of the Interbull dairy evaluation since 2001. Another commonly used strategy for propagating the genomic information to approximate the reliabilities of GEBV for the non-genotyped animals has two steps. First, weights based on the differences between the genomic and conventional reliabilities are derived for the genotyped animals. Second, these weights are used to represent the extra genomic information on the traditional PBLUP [14, 15, 18]. It should be noted that, with the above strategy, the original full PBLUP model has to be reconstructed with the derived weights to obtain the reliabilities of GEBV for the non-genotyped animals. Thus, the same procedure to absorb effects in the coefficient matrix needs to be performed twice, which means doubling the computing time of the PBLUP model. Moreover, double-counting of the genomic information has to be avoided, or at least minimized, when propagating the genomic information into the non-genotyped animals. Therefore, the method for approximating the reliabilities of GEBV for the non-genotyped animals should be optimized to avoid double-counting and enable efficient computation for large-scale datasets.

In this study, we present computational approaches for approximating the reliabilities of GEBV with a single-step model including RPG effects. Specifically, we provide computationally efficient and simplified procedures for both the genotyped and the non-genotyped animals. We also derived and compared different correction terms for the genomic information. The aims of this study were: (1) to describe a multi-step approach for approximating the reliabilities of GEBV with optimized procedures for both the genotyped and non-genotyped animals; (2) to investigate whether the choice of different schemes that propagate the genomic information could affect the accuracy of the reliabilities of GEBV for the non-genotyped animals; and (3) to demonstrate the accuracy and efficiency of the derived methods using two dairy cattle datasets.

## Methods

We present a seven-step method to approximate the reliabilities of GEBV in a single-step model with RPG effects. The first step computes the reliabilities of traditional EBV for all the animals in the pedigree without accounting for genomic information. This is followed by the computation of the reliabilities of GEBV for the genotyped animals (Steps 2 and 3) and then for the non-genotyped animals (Steps 4–7).

### Steps for approximating single-step reliabilities

#### Step 1: reliabilities of EBV using PBLUP

The reliabilities of traditional EBV for all the animals in a PBLUP model can be approximated by many approaches [10, 21] but we used the method of Tier and Meyer [6] to approximate the reliabilities of EBV because it allows for a multi-trait animal model. This step requires phenotypic data, full pedigree information, the model effects from the corresponding single-step model, and its variance components for all random effects to calculate the reliabilities of EBV (${\mathbf{r}}_{p}^{2}$) for all animals and traits. For each trait, the reliabilities are then split into two vectors, i.e., $${\mathbf{r}}_{p}^{2}=\left[\begin{array}{c}{\mathbf{r}}_{p,n}^{2}\\ {\mathbf{r}}_{p,g}^{2}\end{array}\right]$$, where ${\mathbf{r}}_{p,n}^{2}$ and ${\mathbf{r}}_{p,g}^{2}$ are the reliabilities of EBV for the non-genotyped and genotyped animals, respectively.

#### Step 2: reverse reliabilities for the genotyped animals

An iterative procedure called the reverse reliability approach [22] was used to compute the ERC by reversing the method of Tier and Meyer [6] using the reliabilities of the EBV of the genotyped animals from Step 1 (${\mathbf{r}}_{p,g}^{2}$), the full pedigree information, and the variance components. We denoted the resulting ERC for the genotyped animals as ERC_*g*_.

#### Step 3: reliabilities of GEBV for the genotyped animals

In this step, genomic and pedigree information are combined to compute the reliabilities of GEBV for the genotyped animals. Two approaches to account for the RPG effects (see Section “Reliabilities for the genotyped animals”), called the direct method and the blended method are given.

#### Step 4: weights for the non-genotyped animals

The reverse reliability approach [22] was used to calculate the ERC corresponding to the reliabilities ${\mathbf{r}}_{p}^{2}$ of EBV from Step 1. We denote these ERC as ${\mathbf{E}\mathbf{R}\mathbf{C}}_{f}$ and partitioned $${\mathbf{E}\mathbf{R}\mathbf{C}}_{f}= \left[\begin{array}{c}{\mathbf{E}\mathbf{R}\mathbf{C}}_{f,n}\\ {\mathbf{E}\mathbf{R}\mathbf{C}}_{f,g}\end{array}\right]$$, where ${\mathbf{E}\mathbf{R}\mathbf{C}}_{f,n}$ is for the non-genotyped animals (used in Step 7), and ${\mathbf{E}\mathbf{R}\mathbf{C}}_{f,g}$ is for the genotyped animals.

#### Step 5: weights for the genotyped animals

The ERC calculated in this step (${\mathbf{E}\mathbf{R}\mathbf{C}}_{g,g}$) accounts for the genomic information and is used in Step 7 as weights for the genotyped animals. Five different schemes to compute ${\mathbf{E}\mathbf{R}\mathbf{C}}_{g,g}$ are described (see Section "Alternative weights for genotyped animals").

#### Step 6: combining the weights for all the animals

The vector of weights for all the animals is formed by aggregating the ERC values generated from Steps 4 and 5 for the non-genotyped (${\mathbf{E}\mathbf{R}\mathbf{C}}_{f,n}$) and genotyped animals (${\mathbf{E}\mathbf{R}\mathbf{C}}_{g,g}$), respectively. The combined ERC matrix is denoted as $${\mathbf{E}\mathbf{R}\mathbf{C}}_{ss}=\left[\begin{array}{c}{\mathbf{E}\mathbf{R}\mathbf{C}}_{f,n}\\ {\mathbf{E}\mathbf{R}\mathbf{C}}_{g,g}\end{array}\right]$$. In order to avoid possible problems caused by extreme weights, ERC values less than 0.01 were set to 0.01.

#### Step 7: reliabilities of GEBV for the non-genotyped animals

A simplified weighted-PBLUP model with pseudo phenotypes (which can be any real random number since computation of PEV does not require the right-hand side of MME) for all animals in the pedigree was used to obtain the reliabilities of GEBV for the non-genotyped animals. The single-trait weighted-PBLUP model was:

$$\mathbf{y}=\mathbf{1}\mu +\mathbf{a}+\mathbf{e},$$

where $\mathbf{y}$ is a $p$ × 1 vector of pseudo phenotypes with $p$ equal to the number animals in the pedigree; $\mu$ is the general mean; $\mathbf{1}$ is a $p$ × 1 vector of 1s; $\mathbf{a}$ represents a $p$ × 1 vector of additive genetic effects; $\mathbf{e}$ is a vector of residuals. It is assumed that $\mathbf{a} \sim N(\mathbf{0}, \mathbf{A}{\sigma }_{u}^{2})$ and $\mathbf{e} \sim N(\mathbf{0}, {\mathbf{D}}_{p}^{-1}{\sigma }_{e}^{2})$, where $\mathbf{A}$ is the numerator relationship matrix and ${\mathbf{D}}_{p}$ is a diagonal matrix with elements of ERC from Step 6, and ${\sigma }_{u}^{2}$ and ${\sigma }_{e}^{2}$ are the additive genetic and residual variances, respectively.

### Reliabilities for the genotyped animals

Reliabilities for the genotyped animals can be calculated with GBLUP [23] or SNPBLUP [24] in this step. When the number of genotyped animals ($n$) is smaller than the number of SNPs ($m$), a simplified GBLUP model with the RPG effects can be computationally more efficient than SNPBLUP [18]. However, when $n>m$, the SNPBLUP model is often computationally less demanding. Two alternative methods can be used for including the RPG effects (illustrated here using SNPBLUP).

#### Direct method

The single-trait SNPBLUP model with RPG effects is:

1 $$\mathbf{y} = \mathbf{1}\mu +\mathbf{W}\mathbf{u}+\mathbf{e},$$

where $\mathbf{y}$ is an $n$  × 1 vector of (pseudo) phenotypes; $\mu$ is the general mean; $\mathbf{1}$ is an $n$ × 1 vector of 1s; $$\mathbf{W}= \left[\begin{array}{cc}\sqrt{1-\omega }\mathbf{Z}& \sqrt{\omega }{\mathbf{I}}_{n}\end{array}\right]$$, and $\omega$ represents the RPG proportion to the total additive genetic variance, $\mathbf{Z}$ is an $n \times m$ matrix of SNP covariates centered by $- 2{p}_{i}$ and scaled by $\sqrt{2\sum {p}_{i}(1-{p}_{i})}$ using VanRaden’s method 1 [23] (where ${p}_{i}$ is the frequency of the second allele at locus $i$), ${\mathbf{I}}_{n}$ is an identity matrix of order $n$; $$\mathbf{u}= \left[\begin{array}{c}\mathbf{g}\\ \mathbf{a}\end{array}\right]$$, where $\mathbf{g}$ is an $m$ × 1 vector of the effects of SNPs and ${\mathbf{I}}_{n}$ is a $n$ × 1 vector of the RPG effects; $\mathbf{e}$ is a vector of residuals. It is assumed that $\mathbf{a} \sim N(\mathbf{0}, {\mathbf{A}}_{22}{\sigma }_{u}^{2})$, $\mathbf{g} \sim N(\mathbf{0}, {{\mathbf{I}}_{m}\sigma }_{u}^{2})$, and $\mathbf{e} \sim N(\mathbf{0}, {\mathbf{D}}_{n}^{-1}{\sigma }_{e}^{2})$, where ${\mathbf{A}}_{22}$ is the submatrix of $\mathbf{A}$ corresponding to the genotyped animals, ${\mathbf{D}}_{n}$ is a diagonal matrix with element ${d}_{ii}={\mathrm{ERC}}_{g,i}$ equal to the ${\mathrm{ERC}}_{g}$ value for genotyped animal $i$ calculated at Step 2, and ${\sigma }_{u}^{2}$ and ${\sigma }_{e}^{2}$ are the additive genetic and the residual variances, respectively.

The MME for Model (1) is:

$$\left[\begin{array}{cc}{\mathbf{1}}^{^{\prime}}{\mathbf{D}}_{n}\mathbf{1}& {\mathbf{1}}^{^{\prime}}{\mathbf{D}}_{n}\mathbf{W}\\ {\mathbf{W}}^{^{\prime}}{\mathbf{D}}_{n}\mathbf{1}& {\mathbf{W}}^{^{\prime}}{\mathbf{D}}_{n}\mathbf{W}+\lambda {{\varvec{\Omega}}}^{-1}\end{array}\right]\left[\begin{array}{c}\widehat{\mu }\\ \widehat{\mathbf{u}}\end{array}\right]=\left[\begin{array}{c}{\mathbf{1}}^{^{\prime}}{\mathbf{D}}_{n}\mathbf{y}\\ {\mathbf{W}}^{^{\prime}}{\mathbf{D}}_{n}\mathbf{y}\end{array}\right],$$

where $${\varvec{\Omega}}=\left[\begin{array}{cc}{\mathbf{I}}_{m}& \mathbf{0}\\ \mathbf{0}& {\mathbf{A}}_{22}\end{array}\right]$$ and $\lambda =\frac{{\sigma }_{e}^{2}}{{\sigma }_{u}^{2}}$. We denote the inverse of the LHS matrix of the MME as $$\left[\begin{array}{cc}{\mathbf{C}}^{\mu \mu }& {\mathbf{C}}^{\mu \mathbf{u}}\\ {\mathbf{C}}^{\mathbf{u}\mu }& {\mathbf{C}}^{\mathbf{u}\mathbf{u}}\end{array}\right]$$. The reliability of the GEBV for genotyped animal $i$ is ${r}_{g,g,i}^{2}=1- \lambda \frac{{\mathbf{W}}_{i}{\mathbf{C}}^{\mathbf{u}\mathbf{u}}{\mathbf{W}}_{i}^{^{\prime}}}{{{\mathbf{G}}_{\omega }}_{ii}}$, where ${\mathbf{W}}_{i}$ represents row $i$ in $\mathbf{W}$, and ${{\mathbf{G}}_{\omega }}_{ii}$ is the diagonal element $i$ of the matrix ${\mathbf{G}}_{\omega }=\left(1-\omega \right)\mathbf{Z}{\mathbf{Z}}^{^{\prime}}+\omega {\mathbf{A}}_{22}$.

#### Blended method

The single-trait SNPBLUP model without the RPG effects is:

2 $$\mathbf{y}=\mathbf{1}\mu +\mathbf{Z}\mathbf{g}+\mathbf{e},$$

where the same notation and assumptions as in Model (1) are used. Thus, it is assumed that $\mathbf{g} \sim N(\mathbf{0}, {{\mathbf{I}}_{m}\sigma }_{u}^{2})$ and $\mathbf{e} \sim N(\mathbf{0}, {\mathbf{D}}_{n}^{-1}{\sigma }_{e}^{2})$. The MME for Model (2) is:

3 $$\left[\begin{array}{cc}{\mathbf{1}}^{^{\prime}}{\mathbf{D}}_{n}\mathbf{1}& {\mathbf{1}}^{^{\prime}}{\mathbf{D}}_{n}\mathbf{Z}\\ {\mathbf{Z}}^{^{\prime}}{\mathbf{D}}_{n}\mathbf{1}& {\mathbf{Z}}^{^{\prime}}{\mathbf{D}}_{n}\mathbf{Z}+\lambda {\mathbf{I}}_{m}\end{array}\right]\left[\begin{array}{c}\widehat{\mu }\\ \widehat{\mathbf{g}}\end{array}\right]=\left[\begin{array}{c}{\mathbf{1}}^{^{\prime}}{\mathbf{D}}_{n}\mathbf{y}\\ {\mathbf{Z}}^{^{\prime}}{\mathbf{D}}_{n}\mathbf{y}\end{array}\right].$$

We partitioned and denoted the inverse of the LHS matrix of the MME as $$\left[\begin{array}{cc}{\mathbf{C}}^{\mu \mu }& {\mathbf{C}}^{\mu \mathbf{g}}\\ {\mathbf{C}}^{\mathbf{g}\mu }& {\mathbf{C}}^{\mathbf{g}\mathbf{g}}\end{array}\right]$$. The reliability of GEBV for genotyped animal $i$ is ${r}_{g,g,i}^{2*}=1- \lambda \frac{{\mathbf{Z}}_{i}{\mathbf{C}}^{\mathbf{g}\mathbf{g}}{\mathbf{Z}}_{i}^{^{\prime}}}{{\mathbf{G}}_{ii}}$, where ${\mathbf{Z}}_{i}$ represents row $i$ in $\mathbf{Z}$, and ${\mathbf{G}}_{ii}$ is the diagonal element $i$ of the genomic relationship matrix $\mathbf{G}=\mathbf{Z}{\mathbf{Z}}^{^{\prime}}$.

The RPG effects can be accounted for in the final reliability of GEBV by blending the Model (2) reliabilities with the reliabilities of the traditional EBV from PBLUP in Step 1 using the following equation:

$${r}_{g,g,i}^{2}=\frac{\left(1 - \omega \right){\mathbf{G}}_{ii}{r}_{g,g,i}^{2*} + \omega {{\mathbf{A}}_{22}}_{ii}{r}_{p,g,i}^{2}}{\left(1 - \omega \right){\mathbf{G}}_{ii} + \omega {{\mathbf{A}}_{22}}_{ii}},$$

where ${{\mathbf{A}}_{22}}_{ii}$ is the diagonal element $i$ of the ${\mathbf{A}}_{22}$ matrix which is equal to $1+{\mathrm{F}}_{i}$ with ${\mathrm{F}}_{i}$ equal to the pedigree-based inbreeding coefficient of animal $i$.

### Alternative weights for genotyped animals

For the computation of the reliabilities of GEBV for the non-genotyped animals in Step 7, the genomic information is included by using weights for the genotyped animals. To compute the weights, the pedigree and the genomic information need to be separated from the reliabilities of the genotyped animals computed in Step 3. This is because the same information is assigned to an individual and its relatives in the computed reliabilities (Steps 1 and 3), hence the use of reliabilities from Step 3 to compute corresponding ERC values can lead to double-counting of information. However, the original record information without genomics, i.e., ERC_g_, is free from double-counting and independent for each individual. Similarly, it is necessary to calculate a measure of genomic information that does not double count information. Several ways to compute this added information due to genomics can be constructed. The following five approaches present such measures in the order which is expected to result in less double-counting of information.

#### Scheme A

$${ERC}_{g,g}=\lambda \frac{{r}_{g,g}^{2}}{1-{r}_{g,g}^{2}},$$

where ${r}_{g,g}^{2}$ is the reliability of GEBV from Step 3 for genotyped animals. This scheme does not take into account that ${r}_{g,g}^{2}$ also includes pedigree information.

#### Scheme B

$${\mathrm{ERC}}_{g,g}={\mathrm{ERC}}_{f,g}+\lambda \left(\frac{{r}_{g,g}^{2}}{1-{r}_{g,g}^{2}}- \frac{{r}_{p,g}^{2}}{1-{r}_{p,g}^{2}}\right),$$

where ${\mathrm{ERC}}_{f,g}$ is as defined in Step 4, ${r}_{g,g}^{2}$ is as defined in Step 3, and ${r}_{p,g}^{2}$ is the reliability of EBV from Step 1 for the genotyped animals. This scheme is similar to that given in Ben Zaabza et al. [18].

#### Scheme C

$${\mathrm{ERC}}_{g,g}={\mathrm{ERC}}_{f,g}+\lambda \frac{{r}_{g,g}^{2}}{1-{r}_{g,g}^{2}}- {\mathrm{ERC}}_{i,g},$$

where ${\mathrm{ERC}}_{f,g}$ is as defined in Step 4, ${r}_{g,g}^{2}$ is as defined in Step 3, ${\mathrm{ERC}}_{i,g}$ is the ERC value for the genotyped animals calculated using the Interbull method [25] and PBLUP model to account for the information from the genotyped progeny. This approach is similar to Liu et al. [13].

#### Scheme D

$${\mathrm{ERC}}_{g,g}={\mathrm{ERC}}_{f,g}+\lambda \frac{{r}_{g,g}^{2}}{1-{r}_{g,g}^{2}}- {\mathrm{ERC}}_{g},$$

where ${\mathrm{ERC}}_{f,g}$ is as defined in Step 4, ${r}_{g,g}^{2}$ is as defined in Step 3, ${\mathrm{ERC}}_{g}$ is the ERC value calculated in Step 2.

#### Scheme E

$${\mathrm{ERC}}_{g,g}={\mathrm{ERC}}_{f,g}+{\mathrm{ERC}}_{g,g,rev}- {\mathrm{ERC}}_{g},$$

where ${\mathrm{ERC}}_{f,g}$ is as defined in Step 4, ${\mathrm{ERC}}_{g}$ is the ERC value calculated in Step 2, ${\mathrm{ERC}}_{g,g,rev}$ is the ERC value for the genotyped animals calculated using the reliabilities of GEBV from Step 3 (${r}_{g,g}^{2}$), full pedigree, and the reverse reliability approach. It is important to note that all ERC values in this scheme are based on the reverse reliability approach to minimize double-counting of information.

### Data

Two datasets, a small (Data 1) and a large (Data 2) one, were used to assess and compare the methods. Data 1 consisted of 47,124 Finnish Red dairy cows with 305-day milk yield records from lactations one to three. Data 2 was from 341,784 Nordic Jersey dairy cattle with stature records from lactations one to three. In total, 46,914 and 41,897 SNPs were used in the analyses with Data 1 and Data 2, respectively. Table 1 presents the number of genotyped animals, the number of pedigree animals, and the number of animals with records in the first, second, and third lactation for Data 1 (milk yield) and Data 2 (stature). The datasets, model and (co)variances components were supplied by Nordic Cattle Genetic Evaluation (NAV).

**Table 1:** Number of genotyped animals, of animals in the pedigree, and of animals with records in the first, second, and third lactation for Data 1 (milk yield) and Data 2 (stature)

Data set (trait)	Data 1 (milk)			Data 2 (stature)		
Lactation	1	2	3	1	2	3
Animals with records	46,535	35,290	23,780	317,560	30,031	30,483
Genotyped animals^a^	19,757	19,757	19,757	110,876	110,876	110,876
Animals in the pedigree	64,808	64,808	64,808	583,916	583,916	583,916

^a^Total number of genotyped animals across lactations

### Study design and comparison statistics

The reliabilities of GEBV from the direct and blended methods were compared with the exact reliabilities of GEBV from ssGBLUP, for Data 1. The PEV computed by inverting the LHS matrix of the MME for Data 1 were used to calculate the exact reliabilities of GEBV. The comparisons used the following statistics: (1) Pearson’s correlation between the exact reliabilities and the approximate reliabilities; (2) the intercept and slope derived by the regression of the exact reliabilities on the approximate reliabilities; and (3) the mean squared error (MSE) between the exact reliabilities and the approximate reliabilities. The same statistics were applied to assess the quality of the approximate reliabilities of GEBV from the direct and blended methods for the genotyped animals and the five schemes for the non-genotyped animals.

For the genotyped animals in Data 2, Pearson’s correlation between the reliabilities of GEBV from the direct method and the blended method, and the intercept and slope of the regression of the reliabilities of GEBV from the direct method on the blended method were computed. For the non-genotyped animals in Data 2, Pearson’s correlations were computed between the approximate reliabilities of GEBV from the five schemes.

### Computations

In Step 3, a multi-trait GBLUP model was used to compute the reliabilities of GEBV for Data 1, and a single-trait SNPBLUP model for the reliabilities of GEBV for Data 2. It is noteworthy that the reliabilities of EBV used in the blended method for Data 2 have to be computed based on the single-trait PBLUP model. The direct and blended methods were performed and compared for both datasets. A proportion of RPG effects ($\omega )$ of 0.3 was used as in the NAV genetic evaluations. In Step 5, the five described schemes from A to E were used to compute weights for the genotyped animals in Step 7. In Step 7, a multi-trait weighted-PBLUP model was used to compute the reliabilities of GEBV for the non-genotyped animals for both Data 1 and Data 2. All analyses were performed using the MiX99 software package [26, 27] on a server with Intel Xeon Gold 6248 CPU (2.5 GHz) and 1 TB RAM using 10 cores.

## Results

### Reliabilities for the genotyped animals

For Data 1, the mean (SD) reliability of GEBV from the exact method was 0.65 (0.090), 0.62 (0.087), and 0.59 (0.087) for the first, second, and third lactation, respectively. When the multi-step approach with the direct method was used to approximate the reliabilities, corresponding values were 0.65 (0.098), 0.61 (0.096), and 0.59 (0.096) for the first, second, and third lactation, respectively. The multi-step approach with the blended method gave mean reliabilities of 0.68 (0.090), 0.65 (0.086), and 0.63 (0.087) for the first, second, and third lactation, respectively. Thus, the mean reliabilities by the exact and direct methods were close but the blended method had a slightly higher mean. The SD values of the exact and the blended methods were close, but the direct method showed a larger variation in the reliabilities.

**Fig. 1:**
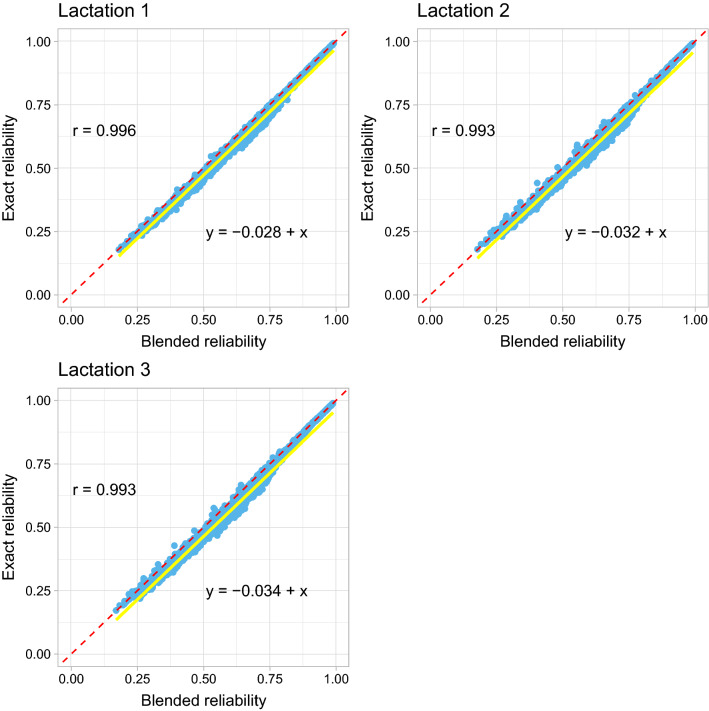
Scatter plot of the reliabilities of genomic estimated breeding values (GEBV) for the genotyped animals by the blended (x-axis) and the exact (y-axis) methods in Data 1 (milk yield). Pearson’s correlation coefficients (r), and linear regression coefficients of the reliabilities of GEBV by the exact method on the blended method are included in the figures. The solid yellow line is the regression line and the dashed red line acts as a reference line with intercept 0 and slope 1

A plot of the reliabilities of GEBV from the exact method versus those from the blended method for Data 1 is in Fig. 1. In general, the correlations between these two methods were close to 1 for all three lactations although a slight decline can be seen from the first to the third lactation (see Fig. 1). The slopes of the linear regression of the reliabilities of GEBV obtained from the exact method on those from the blended method were 1 for all three lactations, but the general mean estimates were slightly less than zero indicating small inflation in the blended reliabilities (Fig. 1). MSE between the reliabilities of GEBV from the exact method and those from the blended method were 0.0009, 0.0013, and 0.0014 for the first, second, and third lactation, respectively.

For the young genotyped animals in Data 1, which were born in the last five years without own phenotypes and progeny, the mean (SD) reliabilities of the GEBV of the genotyped animals from the exact method were 0.57 (0.042), 0.54 (0.043), and 0.52 (0.044) for the first, second, and third lactation, respectively. When the multi-step approach with the direct method was used, corresponding values were 0.57 (0.045), 0.54 (0.045), and 0.52 (0.046) for the first, second, and third lactation, respectively. The multi-step approach with the blended method gave mean reliabilities of 0.60 (0.040), 0.58 (0.040), and 0.56 (0.041) for the first, second, and third lactation, respectively.

**Fig. 2:**
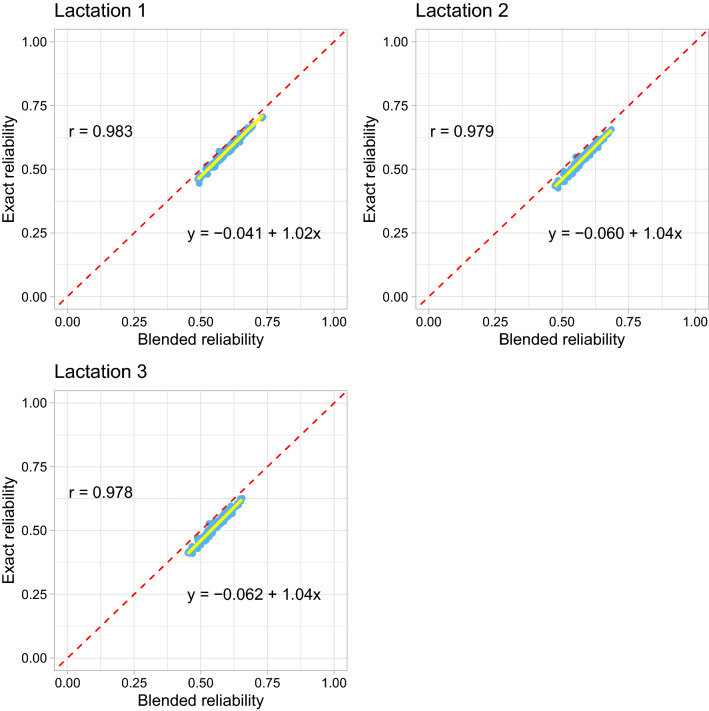
Scatter plot of the reliabilities of genomic estimated breeding values (GEBV) for the young genotyped animals which were born in the last five years without own phenotypes and progeny by the blended (x-axis) and the exact (y-axis) methods in Data 1 (milk yield). Pearson’s correlation coefficients (r), and linear regression coefficients of the reliabilities of GEBV by the exact method on the blended method are included in the figures. The solid yellow line is the regression line and the dashed red line acts as a reference line with intercept 0 and slope 1

Figure 2 shows a similar scatter plot as in Fig. 1 but for the young genotyped animals. The slopes were greater than 1, indicating slight deflation of the approximate reliabilities of GEBV by the blended method for the young genotyped animals.

The approximate reliabilities of GEBV from the direct method agreed well with those obtained by the exact method in Data 1. Compared to the results from the blended method, the correlations between the reliabilities of GEBV from the exact method and those from the direct method (ranging from 0.993 to 0.996) were similar to those using the direct method. The slopes ranged from 0.90 to 0.92 for all the genotyped animals as well as for the young genotyped animals, indicating inflation of the approximate reliabilities of GEBV from the direct method. For all the genotyped animals, MSE between the reliabilities of GEBV from the exact method and those from the direct method were 0.0012, 0.0019, and 0.0020 for the first, second, and third lactation, respectively, which were larger than those from the blended method.

For Data 2, the mean (SD) reliabilities of GEBV for the genotyped animals from the direct method were 0.68 (0.063), 0.49 (0.066), and 0.45 (0.070) for the first, second, and third lactation, respectively. For the blended method, the corresponding values were 0.70 (0.067), 0.51 (0.067), and 0.48 (0.073) for the first, second, and third lactation, respectively. Thus, the average reliability was slightly larger for the blended method than for the direct method but the SD of reliabilities were similar between these two methods. Additional file 1: Table S1 provides the number of animals, mean and SD of the reliabilities of GEBV by birth year for all the genotyped animals using the blended method for three lactations for Data 2.

**Fig. 3:**
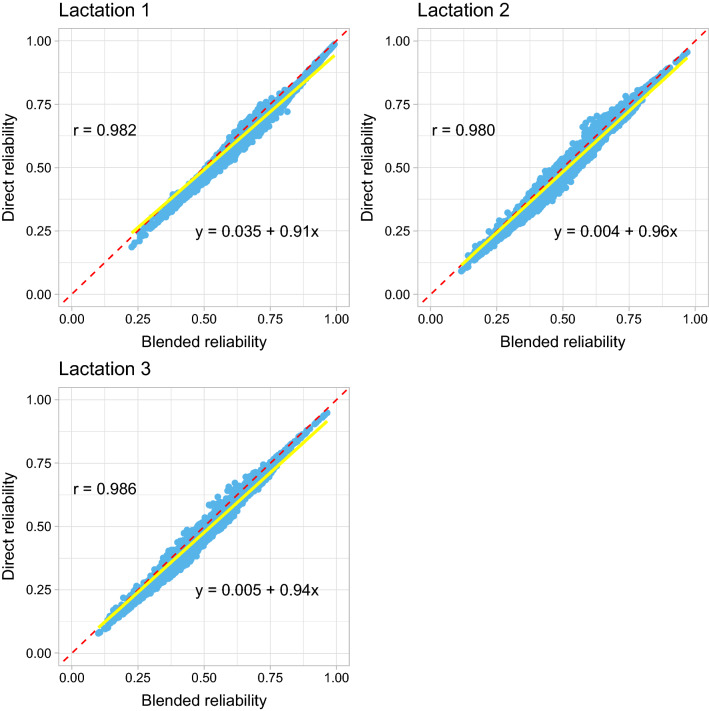
Scatter plot of the reliabilities of genomic estimated breeding values (GEBV) for the genotyped animals by the blended (x-axis) and the direct (y-axis) methods in Data 2 (stature). Pearson’s correlation coefficients (r), and linear regression coefficients of the reliabilities of GEBV from the direct method on the blended method are included in the figures. The solid yellow line is the regression line and the dashed red line acts as a reference line with intercept 0 and slope 1

Figure 3 displays a scatter plot for the reliabilities of GEBV from the direct method versus those from the blended method for the genotyped animals in Data 2. The correlations between the reliabilities of GEBV from these two approximate methods ranged from 0.980 to 0.986. The slopes of the regression of the reliabilities of GEBV from the direct method on those from the blended method were 0.91, 0.96, and 0.94 for the first, second, and third lactation, respectively. In addition, the intercepts were all close to 0.

**Fig. 4:**
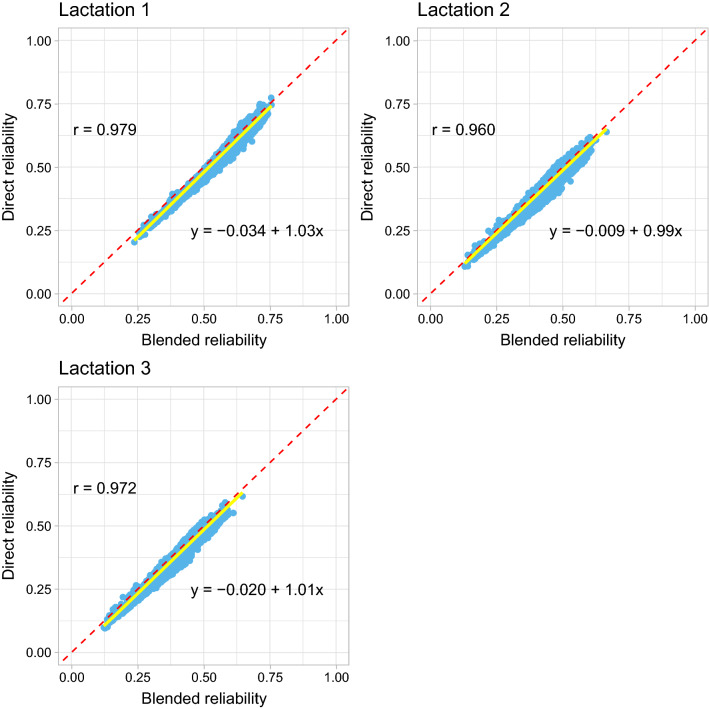
Scatter plot of the reliabilities of genomic estimated breeding values (GEBV) for the young genotyped animals which were born in the last five years without own phenotypes and progeny by the blended (x-axis) and the direct (y-axis) methods in Data 2 (stature). Pearson’s correlation coefficients (r), and linear regression coefficients of the reliabilities of GEBV from the direct method on the blended method are included in the figures. The solid yellow line is the regression line and the dashed red line acts as a reference line with intercept 0 and slope 1

For the young genotyped animals in Data 2, which were born in the last five years without own phenotypes and progeny, the mean (SD) reliabilities of GEBV for the genotyped animals from the direct method were 0.64 (0.050), 0.45 (0.048), and 0.41 (0.047) for the first, second, and third lactation, respectively. For the blended method, the corresponding values were 0.66 (0.047), 0.47 (0.046), and 0.43 (0.045) for the first, second, and third lactation, respectively. Figure 4 shows similar scatter plot as in Fig. 3 but for the young genotyped animals.

For Data 2, the wall clock time to conduct Step 3 using the direct method was approximately 7 h for each trait. The time was reduced to about 30 min using the blended method. The size of MME was 152,774 and peak memory usage was 323 GB for the direct method, whereas the size of MME was 41,898 and peak memory usage was 47 GB for the SNPBLUP model in the blended method.

### Reliabilities for the non-genotyped animals

Tables 2, 3, 4 present statistics for the exact and approximated reliabilities of GEBV for the non-genotyped animals from the different schemes in the analysis of Data 1. The average difference between the approximated and exact reliabilities of GEBV for the non-genotyped animals was small, ranging from − 0.02 to 0.00 for milk yield across three lactations. The results show a decline in the reliability of GEBV from the first lactation to the subsequent lactations, which was expected following the decrease in the number of observations by trait. In general, the approximate reliabilities were equivalent to the exact reliabilities. The correlations between the exact and the approximated reliabilities were slightly lower for subsequent lactations (second and third) compared with those of the first lactation. Similarly, MSE increased slightly as the lactation number increased. The slopes and intercepts of the exact reliabilities on the approximate reliabilities were close to 1 and 0, respectively. As expected, Scheme E yielded the highest correlation between the exact and the approximate reliabilities of GEBV, whereas Scheme A yielded the lowest correlation. This pattern was clearest for lactation 3 (Table 4).

**Table 2:** Mean and standard deviation (SD) of the reliabilities of genomic estimated breeding values (GEBV) calculated by the exact and approximate methods from five schemes

Scheme	Mean(SD)	r	MSE	b_1_	b_0_
Exact^a^	0.47(0.161)				
A	0.46(0.155)	0.991	0.0005	1.03	− 0.01
B	0.46(0.157)	0.993	0.0005	1.02	0.00
C	0.46(0.158)	0.991	0.0006	1.01	0.00
D	0.46(0.155)	0.992	0.0005	1.03	0.00
E	0.45(0.160)	0.994	0.0005	1.00	0.01

Pearson’s correlations (r) and mean squared errors (MSE) between the reliabilities of GEBV, the intercept (b_0_) and slope (b_1_) from the regression of the exact reliabilities on the approximate reliabilities of GEBV for the non-genotyped animals based on Data 1 (milk yield) and lactation 1

^a^Exact reliabilities of GEBV computed by inverting the coefficient matrix of the mixed model equations

**Table 3:** Mean and standard deviation (SD) of the reliabilities of genomic estimated breeding values (GEBV) calculated by the exact and approximate methods from five schemes

Scheme	Mean(SD)	r	MSE	b_1_	b_0_
Exact^a^	0.44(0.150)				
A	0.44(0.146)	0.988	0.0005	1.02	0.00
B	0.43(0.146)	0.991	0.0005	1.01	0.00
C	0.43(0.148)	0.988	0.0007	1.00	0.01
D	0.44(0.146)	0.989	0.0005	1.02	0.00
E	0.43(0.151)	0.992	0.0007	0.99	0.02

Pearson’s correlations (r) and mean squared errors (MSE) between the reliabilities of GEBV, the intercept (b_0_) and slope (b_1_) from the regression of the exact reliabilities on the approximate reliabilities of GEBV for the non-genotyped animals based on Data 1 (milk yield) and lactation 2

^a^Exact reliabilities of GEBV computed by inverting the coefficient matrix of the mixed model equations

**Table 4:** Mean and standard deviation (SD) of the reliabilities of genomic estimated breeding values (GEBV) calculated by the exact and approximate methods from five schemes

Scheme	Mean(SD)	r	MSE	b_1_	b_0_
Exact^a^	0.42(0.144)				
A	0.42(0.140)	0.987	0.0005	1.01	0.00
B	0.41(0.141)	0.990	0.0005	1.01	0.00
C	0.41(0.143)	0.987	0.0007	1.00	0.01
D	0.42(0.141)	0.988	0.0005	1.01	0.00
E	0.41(0.145)	0.992	0.0007	0.98	0.02

Pearson’s correlations (r) and mean squared errors (MSE) between the reliabilities of GEBV, the intercept (b_0_) and slope (b_1_) from the regression of the exact reliabilities on the approximate reliabilities of GEBV for the non-genotyped animals based on Data 1 (milk yield) and lactation 3

^a^Exact reliabilities of GEBV computed by inverting the coefficient matrix of the mixed model equations

For Data 2, the average reliabilities of GEBV ranged from 0.44 to 0.47 for stature across the three lactations. Similarly, as in Data 1, the highest reliabilities were obtained at the first lactation. Tables 5, 6, 7 show the correlations among the five types of reliabilities of GEBV obtained from the different schemes for the non-genotyped animals. Overall, the correlations between the reliabilities were high (around 0.99). High correlations (close to 1) between the reliabilities of GEBV from Schemes A, B, C, and D were observed for each lactation; however, slightly lower correlations (0.994) were found between the reliabilities of GEBV from Scheme E and those from Schemes A, C, and D.

**Table 5:** Pearson’s correlation between the reliabilities of genomic estimated breeding values (GEBV) from different schemes for stature from lactation 1 in Data 2 (stature) for the non-genotyped animals

Scheme	A	B	C	D
B	0.999			
C	1	0.999		
D	1	0.999	1	
E	0.994	0.998	0.995	0.995

**Table 6:** Pearson’s correlation between the reliabilities of genomic estimated breeding values (GEBV) from different schemes for stature from lactation 2 in Data 2 (stature) for the non-genotyped animals

Scheme	A	B	C	D
B	0.998			
C	1	0.998		
D	1	0.998	1	
E	0.994	0.999	0.994	0.994

**Table 7:** Pearson’s correlation between the reliabilities of genomic estimated breeding values (GEBV) from different schemes for stature from lactation 3 in Data 2 (stature) for the non-genotyped animals

Scheme	A	B	C	D
B	0.998			
C	1	0.998		
D	1	0.998	1	
E	0.994	0.999	0.994	0.994

## Discussion

In this paper, we present computationally efficient and simplified procedures to calculate the reliabilities of GEBV. Separate steps were applied for the genotyped and non-genotyped animals. The calculation of reliabilities for the genotyped animals used information from a PBLUP model as weights to the observations in the SNPBLUP or GBLUP model which included the observed genomic information. Likewise, for the non-genotyped animals, the genomic information was included as additional weights for genotyped animals in the weighted-PBLUP model. An important feature of our method is that the models for computing reliabilities (Steps 3 and 7) have only general mean and genetic effects but use different weights and relationship structures. This gives computational simplicity and speed. However, because of the multi-step nature of the approach, it is essential to correctly define the ERC weights used in these models (Steps 3 and 7). The results demonstrated that the reliabilities of GEBV from the single-step method can be accurately and efficiently approximated with the proposed multi-step method.

### Reliability and the residual polygenic effect

When the blended method was applied to approximate the reliabilities of GEBV for the genotyped animals, the dimension of the coefficient matrix of MME in the SNPBLUP model was reduced from $(n+m+1)\times (n+m+1)$ to $(m+1)\times (m+1)$ because the genotyped animals ($n$) representing the polygenic effects were ignored in the model, and the genetic part of the SNPBLUP model only contained the effects of SNPs ($m$). Therefore, the major advantage of the SNPBLUP model is preserved, that is, in spite of the increase in the number of genotyped animals, the dimension of the coefficient matrix of MME remains constant with the number of SNPs. Consequently, the memory used for the MME remains low in the blended approach but grows in squared power of $n$ in the direct method. Furthermore, the computational time is $O({m}^{3})$ instead of $O({(m+n)}^{3})$. This highlights the potential usefulness of the SNPBLUP model and is particularly relevant for large-scale genomic evaluations.

To overcome the computational challenges in the SNPBLUP model with the polygenic effects, Ben Zaabza et al. [19] proposed a Monte Carlo (MC)-based sampling method to approximate the reliability of the SNPBLUP model with the polygenic effects (MC-SNPBLUP), where the dimension of the MME depends on the number of SNPs and MC samples. They reported that the approximate reliabilities of GEBV from the MC-SNPBLUP method were highly correlated with those from the exact method with as few as 5000 MC samples. However, larger MC samples were needed to achieve a good approximation when the trait had a larger proportion of polygenic effects. More recently, Ben Zaabza et al. [20] extended the MC-SNPBLUP approach with full MC sampling to further reduce the computational burden from SNPBLUP with the polygenic effects. They demonstrated lower computational demands, but a tendency to overestimate the reliabilities of individuals with low reliability. Therefore, the number of generated MC samples must be sufficient to ensure sufficient accuracy, while it should be limited to keep the computational costs low.

In practice, reliabilities are useful for animals that are, for example selection candidates, when their genotype becomes available. An advantage of using the blended method is that a precomputed inverse of the coefficient matrix of the MME in the SNPBLUP model from the reference animals can be used. The precomputed PEV matrix of the markers can then be used with new genotypes without the large computational cost needed for making the complete MME and inverting its coefficient matrix. This approach can be used because the information of these new genotypes is not needed in computing the PEV of marker effects. In contrast, when an RPG effect is present in the model, such as in the direct approach, the MME having the reference and the candidate animal genotypes as well as the pedigree information needs to be computed and the coefficient matrix inverted. Thus, the blended method allows a fast approach for computing the reliabilities of GEBV for the new genotypes.

### GBLUP model with polygenic effects

When the number of genotyped animals is smaller than the number of SNPs, as in Data 1 of the current study, the use of a GBLUP model can be preferred over the SNPBLUP model when computing the reliabilities of GEBV. More specifically, an attractive feature of the GBLUP model is that the inclusion of the RPG effects is through a genomic relationship matrix combined with the ${\mathbf{A}}_{22}$ matrix. Therefore, unlike the SNPBLUP model, the dimension of the MME for the GBLUP model remains unchanged. However, with a larger number of genotyped animals, the GBLUP model becomes computationally more burdensome due to the need to invert two dense matrices ($\mathbf{G}$ and the coefficient matrix of MME). A possible alternative, proposed by Bermann et al. [16], is to use the GBLUP model with APY where a sparse inverse of an APY genomic relationship matrix (${\mathbf{G}}_{APY}^{-1}$) is used instead of the original dense ${\mathbf{G}}^{-1}$. The authors showed that the reliabilities of GEBV can be approximated accurately and efficiently with their method. They also showed that their method does not need to construct and invert the MME, but it needs to approximate the weight to be added to the diagonal elements of the inverse of the genomic relationship matrix. Similar to the SNPBLUP approach using the blended method, the ${\mathbf{G}}_{APY}^{-1}$ matrix can be saved in a file and used later in the calculation of the reliabilities of GEBV for the newly genotyped animals.

### A simplified procedure for the non-genotyped animals

To compute the reliabilities of GEBV for the non-genotyped animals, a commonly-used technique is to propagate the genomic information to the non-genotyped relatives [14–16]. The process of propagation generally starts with the computation of the difference between the reliabilities of GEBV and the reliabilities of PBLUP EBV for the genotyped animals. Subsequently, these gained reliabilities are transformed into ERC to add to the conventional PBLUP model. The computation for this step can vary depending on the modeling strategy. For example, Ben Zaabza et al. [18] used a procedure, where the step of computing the reliabilities of PBLUP EBV (analogous to Step 1 in this study) was augmented by extra phenotypes from the genotyped animals for the computation of reliabilities for the non-genotyped animals. The extra phenotypes were weighted with a type of ERC value called ERC_add_, which reflects the differences between the ERC transformed from the reliabilities of GEBV and those from the reliabilities of traditional EBV. A disadvantage of their approach is that the original full PBLUP model for the computation of the reliabilities of EBV (Step 1) needs to be used also in the computation of reliabilities for the non-genotyped animals. This increases the computational complexity because these extra records from the genotyped animals were treated as “repeated records” and augmented to the original PBLUP model. We proposed to use a weighted-PBLUP model for the same task where weights are used to account for the information from the original PBLUP model. The weight in Scheme B uses the same formulas as in their study to combine the pedigree and genomic information for the genotyped animals without the need to use the original PBLUP model.

### Impact of double counting

The computation of the reliabilities of GEBV for the non-genotyped animals using the weighted-PBLUP model in Step 7 attempts to combine information from the original PBLUP (Step 1) and the genomic (Step 3) models by assigning appropriate weights for all animals. These weights are computed using the reverse reliability approach which is expected to account for the original information in the PBLUP model of Step 1. Care is needed to calculate the weights for the genotyped animals to avoid double-counting of information. This is because closely-related animals can use the same information to calculate the EBV and its reliability. For example, when both the sire and its daughter are genotyped, but only the daughter has an observation, the sire EBV and its reliability in PBLUP will include the daughter phenotype information. Therefore, we considered several schemes to compute the ERC values to be used as weights in the weighted-PBLUP model of Step 7.

We hypothesized that Scheme E would achieve the best results whereas Scheme A the worst results. This is because all the ERC values for the genotyped animals in Scheme E (${\mathrm{ERC}}_{g,g}$) were calculated by the reverse reliability approach, whereas the ERC values in Scheme A were simply transformed reliabilities of GEBV for the genotyped animals in Step 3. Scheme E gave the highest correlation with the exact reliabilities for Data 1 and had the lowest correlations with the other schemes for Data 2. The outcome of Scheme B, which produced the second best results, was somewhat unexpected. A possible explanation for this might be that the double-counted information canceled out each other between the ERC of GEBV and the ERC of EBV for the genotyped animals in the term $\lambda (\frac{{r}_{g,g}^{2}}{1-{r}_{g,g}^{2}}- \frac{{r}_{p,g}^{2}}{1-{r}_{p,g}^{2}})$ of Scheme B. In general, the differences between the schemes were negligible such that the amount of double-counting was small in our examples.

We applied the multi-step GEBV reliability approach in analyses of two multi-trait models. A multi-trait GBLUP model was used for the smaller dataset, but a single-trait SNPBLUP was used for the larger dataset. Although our approach was able to compute reliabilities that were highly concordant with the exact reliabilities using a single-trait SNPBLUP model, this may not be optimal. In practice, BLUP is often used in multiple-trait models so that information is shared across genetically correlated traits. Thus, a multi-trait SNPBLUP model might be more appropriate.

Step 3, which includes making and inverting the coefficient matrix in the SNPBLUP model, was the most time-consuming part, while all the other steps were processed in seconds for the datasets in our example. Although this process can be further improved with the saved inverse matrix [28], further investigations should be carried out to assess the impact of using subsets of SNPs or genotyped animals on the quality of the approximated reliabilities of GEBV. Moreover, a large dataset and a complex model can have a large computational load in Step 1, particularly when the number of data records exceeds the number of pedigree records such as in test day models. Thus, the computational needs can depend on the model and data, and have relative costs that differ from those in our examples.

## Conclusions

We present approaches that are suitable to approximate reliabilities from multi-trait ssGBLUP. In these approaches, we propose computationally efficient and simplified procedures for both genotyped and non-genotyped animals. The approximated reliabilities from our approach were in good agreement with the exact reliabilities using the full dataset in a ssGBLUP evaluation. The blended method for the calculation of the reliabilities of GEBV for the genotyped animals is more feasible than the direct method when the RPG effects are accounted for, particularly for large datasets. Scheme E gave the most accurate reliabilities for the non-genotyped animals, followed by Scheme B, with respect to minimizing the double-counting of information. The approach provides an effective strategy for obtaining the reliabilities of GEBV from a ssGBLUP model in practice.

## Supplementary Information

**Additional file 1: Table S1.** Number of animals (N), mean, and standard deviation (SD) of reliabilities of the genomic estimated breeding values (GEBV) by birth year for all the genotyped animals using the blended method for three lactations based on Data set 2.

## References

[1] Meuwissen T, Hayes BJ, Goddard ME (2016). Genomic selection: a paradigm shift in animal breeding. Anim Front.

[2] Legarra A, Aguilar I, Misztal I (2009). A relationship matrix including full pedigree and genomic information. J Dairy Sci.

[3] Christensen OF, Lund MS (2010). Genomic prediction when some animals are not genotyped. Genet Sel Evol.

[4] Mantysaari EA, Koivula M, Stranden I (2020). Symposium review: single-step genomic evaluations in dairy cattle. J Dairy Sci.

[5] Misztal I, Wiggans GR (1988). Approximation of prediction error variance in large-scale animal models. J Dairy Sci.

[6] Tier B, Meyer K (2004). Approximating prediction error covariances among additive genetic effects within animals in multiple-trait and random regression models. J Anim Breed Genet.

[7] Harris B, Johnson D (1998). Approximate reliability of genetic evaluations under an animal model. J Dairy Sci.

[8] Boichard D, Lee AJ (1992). Approximate accuracy of genetic evaluation under a single-trait animal model. J Dairy Sci.

[9] Meyer K (1989). Approximate accuracy of genetic evaluation under an animal model. Livest Prod Sci.

[10] Liu Z, Reinhardt F, Bunger A, Reents R (2004). Derivation and calculation of approximate reliabilities and daughter yield-deviations of a random regression test-day model for genetic evaluation of dairy cattle. J Dairy Sci.

[11] Misztal I, Tsuruta S, Aguilar I, Legarra A, VanRaden PM, Lawlor TJ (2013). Methods to approximate reliabilities in single-step genomic evaluation. J Dairy Sci.

[12] Fernando RL, Dekkers JCM, Garrick DJ (2014). A class of Bayesian methods to combine large numbers of genotyped and non-genotyped animals for whole-genome analyses. Genet Sel Evol.

[13] Gao H, Koivula M, Jensen J, Stranden I, Madsen P, Pitkanen T (2018). Short communication: genomic prediction using different single-step methods in the Finnish red dairy cattle population. J Dairy Sci.

[14] Liu Z, VanRaden PM, Lidauer MH, Calus MP, Benhajali H, Jorjani H (2017). Approximating genomic reliabilities for national genomic evaluation. Interbull Bull.

[15] Edel C, Pimentel ECG, Erbe M, Emmerling R, Gotz KU (2019). Short communication: calculating analytical reliabilities for single-step predictions. J Dairy Sci.

[16] Bermann M, Lourenco D, Misztal I (2022). Efficient approximation of reliabilities for single-step genomic best linear unbiased predictor models with the algorithm for Proven and Young. J Anim Sci.

[17] Misztal I (2016). Inexpensive computation of the inverse of the genomic relationship matrix in populations with small effective population size. Genetics.

[18] Ben Zaabza H, Taskinen M, Mantysaari EA, Pitkanen T, Aamand GP, Stranden I (2022). Breeding value reliabilities for multiple-trait single-step genomic best linear unbiased predictor. J Dairy Sci.

[19] Ben Zaabza H, Mäntysaari EA, Strandén I (2020). Using Monte Carlo method to include polygenic effects in calculation of SNP-BLUP model reliability. J Dairy Sci.

[20] Ben Zaabza H, Mäntysaari EA, Strandén I (2021). Estimation of individual animal SNP-BLUP reliability using full Monte Carlo sampling. JDS Commun.

[21] Ducrocq V, Schneider MP (2007). Generalization of the information source method to compute reliabilities in test models. Interbull Bull.

[22] Taskinen M, Mäntysaari EA, Aamand GP, Strandén I. Comparison of breeding values from single-step and bivariate blending methods. In proceedings of the 10th world congress on genetics applied to livestock production: 18–22 August 2014; Vancouver; 2014.

[23] VanRaden PM (2008). Efficient methods to compute genomic predictions. J Dairy Sci.

[24] Strandén I, Garrick DJ (2009). Technical note: derivation of equivalent computing algorithms for genomic predictions and reliabilities of animal merit. J Dairy Sci.

[25] Strandén I, Lidauer M, Mäntysaari EA, Pösö J (2000). Calculation of interbull weighting factors for the Finnish test day model. Interbull Bull.

[26] Strandén I, Lidauer MH (1999). Solving large mixed linear models using preconditioned conjugate gradient iteration. J Dairy Sci.

[27] Pitkänen PJ, Gao H, Kudinov A, Taskinen M, Mäntysaari EA, Lidauer MH, Strandén I. From data to genomic breeding values with the MiX99 software suite. In proceedings of the 12th world congress on genetics applied to livestock production: 3–8 July 2022; Rotterdam; 2022.

[28] Ben Zaabza H, Mäntysaari EA, Strandén I (2020). Snp_blup_rel: software for calculating individual animal SNP-BLUP model reliabilities. Agric Food Sci.

