# Supplementary material for: A computationally efficient method for approximating reliabilities in large-scale single-step genomic prediction
Source: Genet Sel Evol. 2023 Jan 5;55:1. doi: 10.1186/s12711-022-00774-y (PMC9814342; doi:10.1186/s12711-022-00774-y)
Supplement: Supplementary file 1 — Additional file 1: Table S1. Number of animals (N), mean, and standard deviation (SD) of reliabilities of the genomic estimated breeding values (GEBV) by birth year for all the genotyped animals using the blended method for three lactations based on Data set 2. [file 12711_2022_774_MOESM1_ESM.docx]

**Table S1** Number of animals (N), mean, and standard deviation (SD) of reliabilities of the genomic estimated breeding values (GEBV) by birth year for all the genotyped animals using the blended method for three lactations based on Data set 2

|  |  | Lactation 1 | | Lactation 2 | | Lactation 3 | |
| --- | --- | --- | --- | --- | --- | --- | --- |
| Birth year | N | Mean | SD | Mean | SD | Mean | SD |
| 2005 | 238 | 0.80 | 0.087 | 0.54 | 0.136 | 0.50 | 0.153 |
| 2006 | 263 | 0.80 | 0.089 | 0.54 | 0.135 | 0.51 | 0.151 |
| 2007 | 284 | 0.81 | 0.082 | 0.57 | 0.141 | 0.54 | 0.155 |
| 2008 | 341 | 0.79 | 0.082 | 0.58 | 0.118 | 0.55 | 0.129 |
| 2009 | 426 | 0.77 | 0.080 | 0.60 | 0.091 | 0.58 | 0.090 |
| 2010 | 2598 | 0.76 | 0.047 | 0.57 | 0.080 | 0.56 | 0.082 |
| 2011 | 4723 | 0.75 | 0.045 | 0.57 | 0.072 | 0.55 | 0.073 |
| 2012 | 5398 | 0.75 | 0.047 | 0.57 | 0.063 | 0.55 | 0.067 |
| 2013 | 4423 | 0.74 | 0.050 | 0.56 | 0.060 | 0.54 | 0.061 |
| 2014 | 5531 | 0.74 | 0.058 | 0.55 | 0.058 | 0.53 | 0.062 |
| 2015 | 6755 | 0.73 | 0.072 | 0.54 | 0.062 | 0.52 | 0.066 |
| 2016 | 8048 | 0.72 | 0.075 | 0.53 | 0.060 | 0.51 | 0.065 |
| 2017 | 10894 | 0.71 | 0.068 | 0.52 | 0.055 | 0.50 | 0.059 |
| 2018 | 12408 | 0.72 | 0.054 | 0.52 | 0.045 | 0.47 | 0.047 |
| 2019 | 15005 | 0.71 | 0.052 | 0.49 | 0.043 | 0.45 | 0.042 |
| 2020 | 17976 | 0.67 | 0.047 | 0.47 | 0.040 | 0.43 | 0.040 |
| 2021 | 14751 | 0.63 | 0.036 | 0.44 | 0.034 | 0.41 | 0.034 |
| 2022 | 814 | 0.62 | 0.032 | 0.43 | 0.031 | 0.40 | 0.031 |
